# Microbial Diversity and Potential Pathogens in Ornamental Fish Aquarium Water

**DOI:** 10.1371/journal.pone.0039971

**Published:** 2012-09-06

**Authors:** Katherine F. Smith, Victor Schmidt, Gail E. Rosen, Linda Amaral-Zettler

**Affiliations:** 1 Department of Ecology and Evolutionary Biology, Brown University, Providence, Rhode Island, United States of America; 2 The Josephine Bay Paul Center for Comparative Molecular Biology and Evolution, Marine Biological Laboratory, Woods Hole, Massachusetts, United States of America; 3 Columbia University Center for Infection and Immunity, Mailman School of Public Health, New York, New York, United States of America; 4 Department of Geological Sciences, Brown University, Providence, Rhode Island, United States of America; University of São Paulo, Brazil

## Abstract

Ornamental fishes are among the most popular and fastest growing categories of pets in the United States (U.S.). The global scope and scale of the ornamental fish trade and growing popularity of pet fish in the U.S. are strong indicators of the myriad economic and social benefits the pet industry provides. Relatively little is known about the microbial communities associated with these ornamental fishes or the aquarium water in which they are transported and housed. Using conventional molecular approaches and next generation high-throughput amplicon sequencing of 16S ribosomal RNA gene hypervariable regions, we characterized the bacterial community of aquarium water containing common goldfish (*Carassius auratus*) and Chinese algae eaters (*Gyrinocheilus aymonieri*) purchased from seven pet/aquarium shops in Rhode Island and identified the presence of potential pathogens. Our survey identified a total of 30 phyla, the most common being Proteobacteria (52%), Bacteroidetes (18%) and Planctomycetes (6%), with the top four phyla representing >80% of all sequences. Sequences from our water samples were most closely related to eleven bacterial species that have the potential to cause disease in fishes, humans and other species: *Coxiella burnetii, Flavobacterium columnare, Legionella birminghamensis, L. pneumophila, Vibrio cholerae*, *V. mimicus*. *V. vulnificus, Aeromonas schubertii*, *A. veronii*, *A. hydrophila* and *Plesiomonas shigelloides*. Our results, combined with evidence from the literature, suggest aquarium tank water harboring ornamental fish are an understudied source for novel microbial communities and pathogens that pose potential risks to the pet industry, fishes in trade, humans and other species.

## Introduction

Ornamental fishes are the third most common group of pets in United States (U.S.) homes today. The 2011–2012 survey of the American Pet Products Manufacturers Association reported that 62% of U.S. households (73 million homes) own a pet. Of these, 17% own ornamental aquarium fishes, totaling 73 million homes with more than 151.1 million freshwater and 8.61 million saltwater fishes. During the past decade, fishes were one of the fastest growing categories of pets in the U.S., increasing in ownership by more than 20% over the previous decade [1]. Ornamental fishes sold in the country are both bred domestically and imported from abroad [2], [3]. More than 90% of live non-domesticated wildlife imported to the U.S. during the period 2000–2006 was freshwater and marine ornamental fishes, originating largely from Southeast Asia, and totaling ∼1.1 billion individuals. On average, ∼18 thousand shipments and ∼187 million live aquarium fishes were imported annually, 99% of which were intended for commercial sale in the pet industry.

The pet industry provides many economic and social benefits and the global scope, scale and growing popularity of the ornamental fish trade are a testament to this. Unintended outcomes can occur, however, including the spread of potential pathogens that may cause disease in trade animals themselves or to other susceptible hosts encountered in supply chains, at pet shops, or end destination aquaria. In particular, carriage and aquarium tank water associated with ornamental fishes provide prime conditions for bacterial growth; most fishes in trade are tropical in origin [2] and require the same warm, nutrient-rich, and aerated environments that favor bacterial growth. To date, very few studies have characterized the overall microbial communities or potential pathogens associated with ornamental fishes or their water [4]–[6]. This was the primary goal of our study.

New molecular strategies introduced by an international effort to census marine life [7]–[10] have enabled rapid and cost effective means of characterizing microbial communities in a range of habitats beyond the marine environment, including human microbiomes [11]–[13], those of other animals [14], [15], and high human impact environments such as waste water and urban air [16], [17]. Like humans, ornamental fishes should possess an order of magnitude more microbial cells than fish cells in their bodies [18]. Characterizing the microbial communities and pathogenic taxa associated with the ornamental fish trade would broadly benefit the aquarium industry, aquaculture, and public health officials concerned with opportunistic bacterial infections in compromised populations. Using a combination of traditional molecular approaches and next generation high-throughput amplicon sequencing of 16S ribosomal RNA gene hypervariable regions, we report results from a survey of the bacterial community composition, a more targeted survey of *Vibrio* and gamma-proteobacterial species composition, and specific potential pathogens found in ornamental fish aquarium tank water at seven pet/aquarium shops in Rhode Island. To our knowledge this is the first study to use high-throughput sequencing methods to characterize the microbial community associated with ornamental fish aquarium water in the pet industry.

## Results

### Microbial Diversity

Our sequencing of bacterial V3–V5 hypervariable regions of the 16S rRNA gene from two aquaria samples, each across three pet/aquarium stores, generated a total of 64,757 reads (mean 10,792 per sample, range 6,934–14,295). We sequenced the same six samples, plus one additional sample from a 4^th^ store, using primers targeting *Vibrio* species spanning the V4 hypervariable region that generated an additional 44,713 16S rRNA gene amplicon reads (mean 7,452 per sample) (Table 1). The latter primers combined a forward general primer (518F) with a *Vibrio*-specific reverse primer and had the advantage of recovering both *Vibrio* species, as well as other members of the Gammaproteobacteria known to harbor potentially pathogenic species.

**Table 1:** Samples collected from bag water harboring *Carassius auratus* (Common Goldfish) or *Gyrinocheilus aymonieri* (Chinese Algae Eater), purchased from four Rhode Island pet stores, and associated run results.

	Sample Collection Information			341F-926R (V3–V5) Amplicon Run*		518F-680R (V4) Amplicon Run*	
Sample name**	Target species	Other species within tank	No. target/No. other	No. reads	Obs. OTUs	No. reads	Obs. OTUs
**A1**	*C. auratus*	*Siluriformes* sp.	19/3	9232	1312	6938	26
**A2**	*G. aymonieri*	*Macropodus opercularis*	14/42	11221	1664	7096	14
		*Danio rerio*					
		*Ancistrus* sp.					
**D1**	*C. auratus*	None	10/0	10268	580	7565	33
**D2**	*G. aymonieri*	*Paracheirodon innesi*	10/20	6934	380	7599	29
**E1**	*C. auratus*	*Hemigrammus ocellifer*	10/1	12807	917	7051	30
**E2**	*G. aymonieri*	*Poecilia reticulata*	2/14	14295	1242	8464	61
		*Corydoras* sp.					
		*Misgurnus anguillicaudatus*					
**B2**	*G. aymonieri*	None	10/0	N/A	N/A	8202	44

* The 341F-926R primers were designed to capture overall bacterial diversity, whereas the 518F-680R primers were designed specifically to capture *Vibrio* diversity.

** Sample names consist of a store ID letter, followed by sample number taken at that store. Only a single sample exists from store B which was included only on the 518F-680R *Vibrio*-targeted amplicon run.

**Figure 1:**
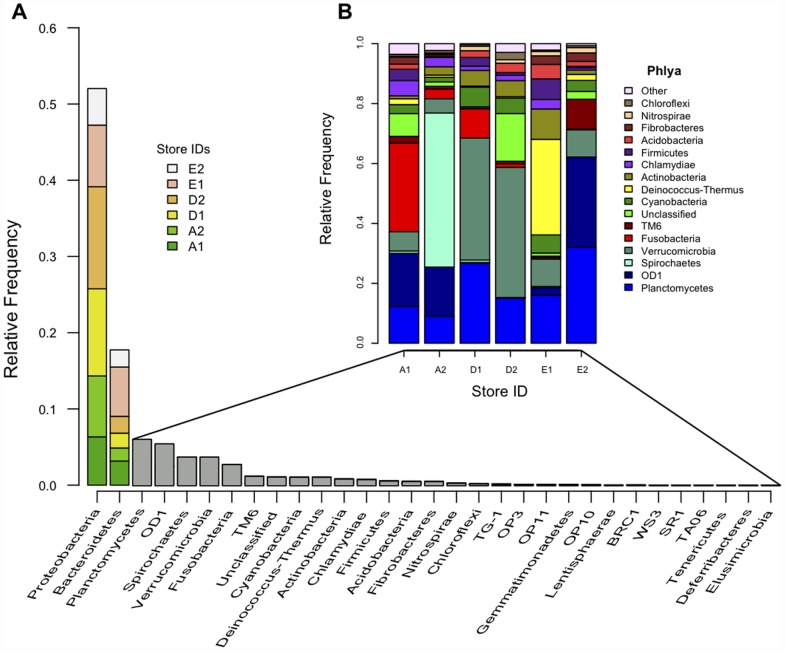
Phylum-level Bacterial Diversity. Phylum-level bacterial diversity as revealed by pyrotag sequencing of the hypervariable V3–V5 region of 16S rRNA genes in our study samples (Table 1). Thirty phyla were detected. **A**) Relative frequency of phyla as a proportion of total tags. Interstore variance in relative frequency is depicted by color for the two most abundant phyla, Proteobacteria and Bacteroidetes, representing ∼70% of all sequences. **B**) Interstore variance in relative frequency for the remaining phyla (<30% of total reads) normalized to 100% after subtracting the Proteobacteria and Bacteriodetes.

Taxonomic analysis of the V3–V5 16S rRNA gene amplicon reads yielded a total of 30 phyla across all samples with the two most abundant, Proteobacteria (mean 51.8%) and Bacteriodetes (mean 17.6%), accounting for nearly 70% of all reads. Several phyla were extremely rare. Elusimicrobia, Deferribacteres and Tenericutes contained only a single read (<0.01%), and WS3, SR1 and TAO6 contained fewer than 10 reads each (<0.03%), despite being present in multiple samples (Figure 1).

**Figure 2:**
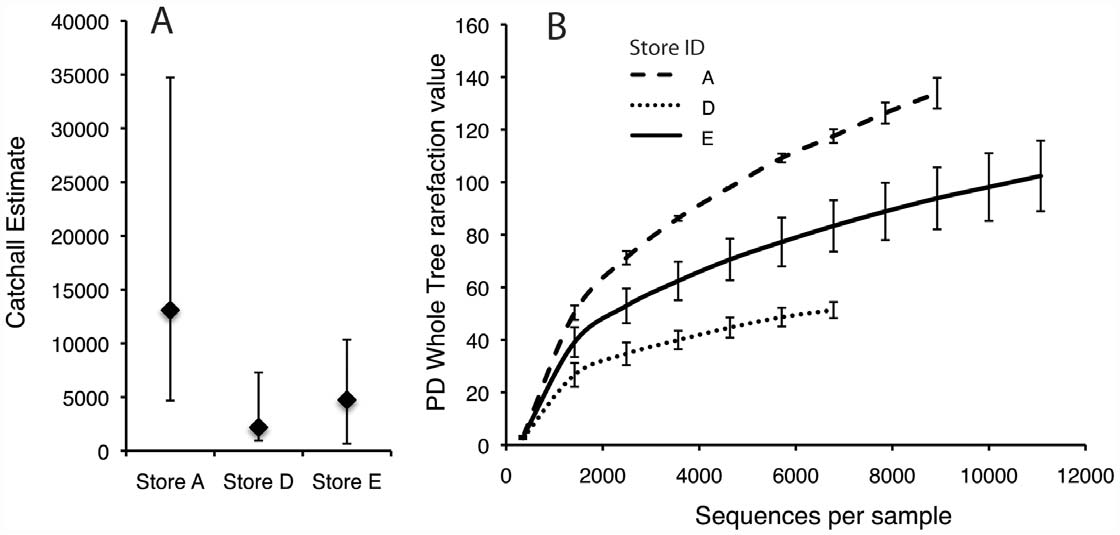
Alpha Diversity in Pet Shops. Alpha diversity within retail stores based on **A**) CatchAll's Best Model analysis with non-rarified data shown with 95% Bonferroni-corrected upper and lower confidence bounds for each estimate. **B**) Phylogenetic Diversity (PD) - Whole Tree analysis calculated after rarifying samples to equal sequencing depth in QIIME. Both metrics were applied to calculate inter-store alpha diversity by grouping 2 samples from each store for a single analysis.

Alpha diversity (within sample diversity) based on species richness estimation from our V3–V5 rRNA gene amplicon sequencing differed significantly and was higher between store A and both stores D and E, but not between stores D and E. Analyses using both phylogeny-based metrics (Phylogenetic Diversity (PD) Whole Tree) as implemented in Qiime v1.4.0 [19] and model-based parametric richness estimated using the CatchAll program [20] showed similar trends in comparative richness between stores (Figure 2). CatchAll estimates from store A were highest with 9,130 estimated species, followed by store E with 5,308 and store D with 1,414 estimated species (Figure 2). Even using the lowest confidence bound for store A (LB = 6,494) and the upper bound for Store D (UB 1,650) this represented a near four-fold difference in bacterial diversity between stores. We found no significant differences in alpha diversity when samples were grouped by the dominant fish species occupying tanks from which samples were collected (data not shown), suggesting inter-store variation had a stronger effect on bacterial diversity than fish species.

**Figure 3:**
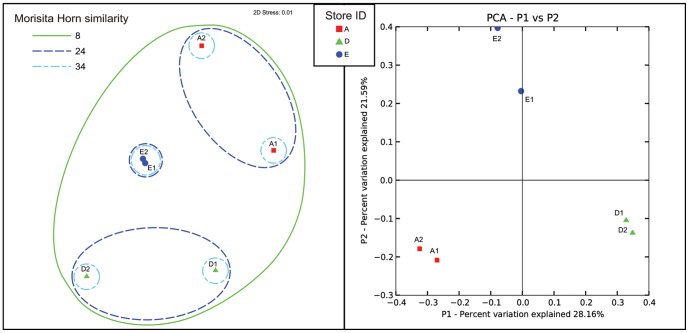
Beta Diversity Between Samples. Beta diversity between samples based on **A**) Morisita-Horn similarity metrics of non-rarified data visualized using Non-Metric Multidimensional Scaling (NMDS). **B**) Unwieghted UNIFRAC distance calculated after rarifying samples to equal sequence depth in QIIME, visualized using Principal Component Analysis.

Beta diversity metrics also showed strong groupings of samples taken from the same store but were not statistically significant (ANOSIM, p = 0.067). UNIFRAC distances [21] (a phylogenic-based, taxonomy-independent metric) between samples within a store were always smaller than any between-store comparisons, and PCA analysis of these distances showed that samples from each store clustered together (Figure 3). This pattern was also evident using OTU abundance-based distance methods, and some degree of clustering was evident with the Bray-Curtis (data not shown) and Morisita-Horn (Figure 3) metrics, although groupings between stores A and D fell apart using Morisita-Horn (Figure 3). These differences are potentially due to heterogeneity in sequence depth between samples (Store A: 20,295, Store D: 16,903, Store E: 27,016).

### PCR Screening and Cloning of Potential Pathogens

We tested for the direct presence of 12 known bacterial or eukaryotic potential pathogens in samples from all seven stores using specific primer sets. Five of the twelve genera (∼42%) were not detected in any of our samples: *Salmonella*, *Giardia, Naegleria*, *Francisella*, and *Campylobacter. Acanthamoeba*, a free-living opportunistic pathogenic amoeba often harboring potentially pathogenic symbiotic bacteria, was ubiquitous across all samples tested (N = 10). The genera *Vibrio, Legionella* and *Mycobacterium* were all found in a minimum of 10 of the 14 tanks from which we sampled, while *Cryptosporidium, Corynebacterineae* and *Aeromonas* were less frequently detected (in 3, 7 and 7 tanks respectively).

It is important to note that a negative PCR result, even after multiple attempts, does not prove the absence of a given species. Despite the use of positive controls in PCR reactions, reasons for false negatives are numerous, including low target abundances, lack of optimized PCR reaction conditions for particular genomic DNA extractions, poor genomic DNA quality, or other methodological factors that may have prevented successful amplification of a given sample. Therefore we did not perform statistical analyses on inter-store differences.

We cloned and sequenced positive PCR amplicons from *Legionella*, *Vibrio*, and *Aeromonas* genus-specific reactions to obtain more refined taxonomic assignments of potential pathogens. Double stranded sequencing of cloned products confirmed the presence of the potential human pathogens *Vibrio vulnificus, V. cholerae, Legionella pneumophila* and *Aeromonas hydrophila* in tank E1.

From the same freshwater aquaria we also sequenced 93 PCR products targeting the near full-length 16S rRNA gene (*E. coli* positions 27 to 1492), to compare these relative abundances with those based on 454 sequencing. In this analysis, only 5 phyla were uncovered, with the most abundant being Proteobacteria (60%), followed by Fusobacteria (27%), Bacteroidetes (12%), Spirochaetes (1%) and Nitrospirae (1%). Surprisingly, 4 potential human or fish pathogen genera were also detected, including *Aeromonas, Flavobacterium, Plesiomonas*, and *Vibrio*.

### *Vibrio* Diversity

**Figure 4:**
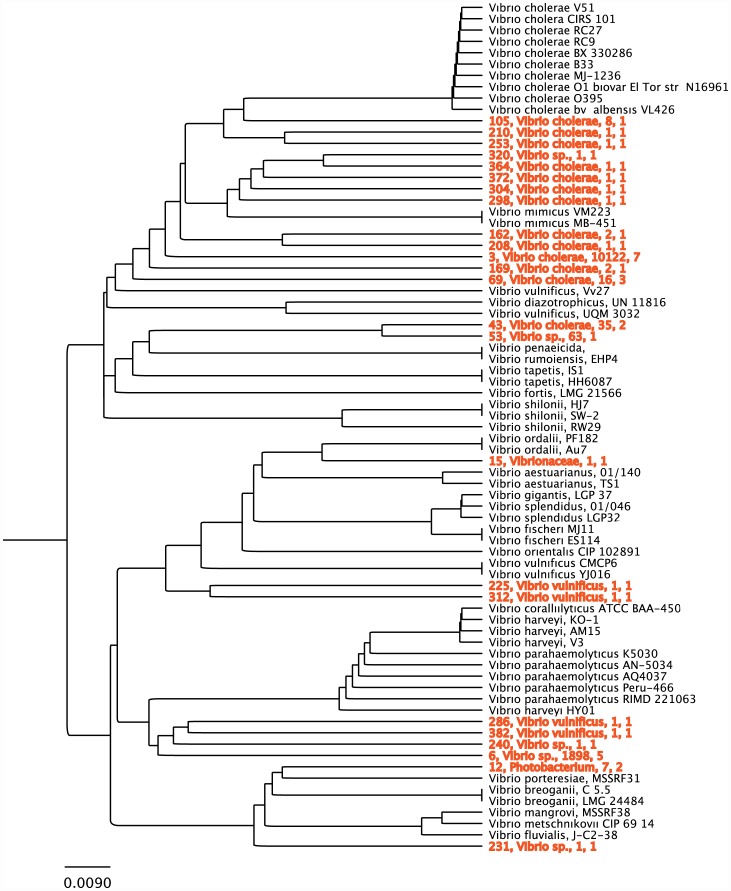
*Vibrio* Reference Tree. *Vibrio* reference tree created using sequences of isolates from the SILVA-ARB 16S rRNA gene database. Shown in red are *Vibrio* OTUs from our FLX run whose GAST taxonomy fell within the *Vibrio* genus. Red OTUs are labeled with species designations, tag count and the number of samples that contained that OTU.

Our targeted *Vibrio* amplicon experiment yielded 27 different *Vibrio* OTUs overall with both samples from store A having only one OTU each, and with the highest number of OTUs coming from Store E and represented by 11 different *Vibrio* OTU types. The phylogenetic placement of these OTUs in a pruned version of the SILVA ARB 5.1 tree is shown in Figure 4. Of all the GAST associated taxonomic assignments, nearly half were assigned to *V. cholerae*, and the remainder were either assigned to *Vibrio* sp. or *V. vulnificus*. However, because GAST takes a very conservative approach to assigning taxonomy it is helpful to examine the relationship between known strains or species of *Vibrio* in the ARB reference tree.

A *Vibrio cholerae* OTU (minimum GAST distance = 0, maximum GAST distance = 1.3%) identified in ARB as strain PIM9 (GenBank #: GQ359963) was dominant in all stores. This OTU ranged from being the only *Vibrio* present (100% of the *Vibrio* community) to 13% of the community. The second most common *Vibrio* OTU was very closely related to a *Vibrio* sp. cultured from a biofilm at a fish farm [22]. In one of our samples this OTU was dominant, constituting 87% of the *Vibrio* community. The remaining *Vibrio* OTUs were very rare – constituting less than 1% of the community membership.

## Discussion

### Microbial Diversity

To the best of our knowledge this is the first survey to characterize the microbiome of water associated with freshwater ornamental aquarium fishes in the pet industry using high-throughput methods. Two earlier studies by Raja et al. [5] and Sugita et al. [6] focused on freshwater filter systems and marine aquaria water respectively, but employed more classical microbiological approaches including Sanger sequencing, bacterial counts and culturing. In these studies the authors recovered only three phyla from marine tanks (Proteobacteria, Bacteroidetes and Firmicutes) and five phyla from freshwater tanks (Proteobacteria, Bacteroidetes, Firmicutes, Nitrospira and Actinobacteria). These results are similar to our freshwater aquaria clone library that recovered 5 phyla (Proteobacteria, Fusobacteria, Bacteroidetes, Spirochaetes and Nitrospirae). In contrast, our high-throughput methods yielded 30 bacterial phyla. This striking contrast illustrates the utility of high-throughput technology to characterize extremely rare but important members of bacterial assemblages; the potential pathogens uncovered in this study are a good example (Table 2).

**Table 2:** Bacterial species identified in this study that have been reported elsewhere as pathogenic in various hosts.

GAST Identified OTU Taxonomy	Number of Positive Samples	Primary Carrier Hosts & Environments*	Hosts that Acquire the Disease	Disease Manifestation	Primary Transmission Route
Sequences matching pathogenic species found with 454 sequenced 341F-926R universal bacterial V3V5 amplicons					
*Coxiella burnetii (1 OTU)*	2	Isolated from aquatic environments, Domestic mammals, Birds [53]	Humans, Livestock, Other domestic mammals [53]–[54]	Q Fever (Humans), Respiratory disease and Abortion (Livestock)	Spore inhalation [53]–[54]
*Flavobacterium columnare (2 OTUs)* **	4	Isolated from aquatic environments [55]–[56] Freshwater fish [55], Saprophyte [56]	Freshwater fish [55]	Columnaris	Contaminated water [56]
*Legionella pneumophila* (2 OTUs)	1	Freshwater amoebae [57], Soils [58]	Humans [59], Protozoa [57]	Legionaire's disease, Pontiac fever (Humans)	Spore inhalation [59]
*Legionella birminghamensis* (2 OTUs)	1	Unknown (presumed freshwater amoebae, soil) [60]	Rare in humans [61]	Pneumonia	Spore inhalation [61]
*Vibrio cholerae* (4 OTUs)**	4	Isolated from aquatic environments, Zooplankton [62], Insects [63], Marine fish, Shellfish [64]	Humans [62], Marine fish [35]	Cholera (Humans), Septicaemia (Fish)	Contaminated water and food [62], [65]
*Vibrio mimicus* (1 OTU)	1	Isolated from aquatic environments [66], Zooplankton, Crustaceans,Reptile eggs [67]	Rare in humans [67]	Diarrhea	Contaminated water and food [62], [65]
Sequences matching pathogenic species found using 454 sequenced 518F-680R *Vibrio* specific V4 primers amplicons					
*Vibrio cholerae* (12 OTUs)	7	See above			
*Vibrio vulnificus* (3 OTUs)	1	Isolated from aquatic environments, Shellfish [68]	Humans [69], Fish, Eels [70]	Wound infections (Humans), Septicemia (Fish)	Contaminated water and food [62], [65]
*Aeromonas schubertii* (3 OTUs)	2	Unknown	Human [21]	Intestinal infection (Humans)	Contaminated water and food [62], [65]
Sequences matching pathogenic species found using full length Sanger sequenced 8F-1492R amplicons					
*Aeromonas veronii* ++	N/A	Isolated from aquatic environments, Medical Leech symbiont [71]	Human [71], [26], Fish [35]	Diarrhea (Humans), Epizootic ulcerative syndrome (Fish)	Contaminated water and food [71], [72]
*Aeromonas hydrophila* ++	N/A	Isolated from aquatic environments [71]	Human [37], [73], Freshwater fish [35], Nematode [71]	Diarrhea (Human), Haemorrhagic septicaemia, Fin rot (Fish)	Contaminated water and food [71], [72]
*Plesiomonas shigelloides*	N/A	Isolated from terrestiral and aquatic environments, Fish, Reptiles, Birds, Mammals [73]–[75]	Humans [69], [73], Freshwater fish [35]	Intestinal infection, diarrhea (Humans), Isolated from morbid individuals (Fish)	Contaminated water and food [72]

* Many of these species have been isolated from environmental samples (eg. aquatic or terrestrial), this does not however imply that they are actively dividing outside of a host.

** These species were also found using full-length 16S rRNA gene Sanger sequencing (see methods).

++ Although these species were not found in either pyrosequencing run, the *Aeromonas* genus was found at high levels. The greater resolution of the longer Sanger reads likely allowed for species level classification with Sanger reads, but not pyrotag reads.

It is difficult to make direct comparisons of our high-throughput results, but in typical freshwater environments (e.g. lakes, streams etc.) Actinobacteria dominate [23] versus the Proteobacteria that dominated our samples (All: 52%, *Aquicella* sp.: 3.8%, *Polynucleobacter cosmopolitanus*: 3%, *Novosphingobium* sp.: 2.6%, *Naxibacter* sp.: 2.5%, *Aeromonas* sp.: 2%). A closer comparative environment for our purposes may be tap water, since freshwater fish tanks in pet/aquarium shops are generally filled using treated tap water. Although no published tap water studies are currently available, the VAMPS (http://vamps.mbl.edu) website has a collection of 25 tap water samples from Falmouth, MA. In comparison to these data (mean relative abundance of tap water), our aquarium water samples had smaller populations of Verrucomicrobia and Cyanobacteria and larger populations of Bacteroidetes, OD1, Fusobacteria and Spirochaetes. Proteobacteria was by far the most abundant phylum in both tap water (64%) and aquarium water (52%). We refrain from making direct comparisons or stating statistically significant differences given that these datasets were collected for different projects and used a different region of the 16S rRNA gene to assign taxonomy to each read, although both did use the GAST taxonomic identification pipeline.

Alpha diversity estimates were similar to those reported in the literature but these vary greatly depending on the freshwater environment, level of impact on the environment, and the contribution of landscape transfers such as soil to the overall community structure [24]. No landscape transfer into or out of our sample tanks was occurring, and their bacterial community structure should have only been a product of the water used to fill the tanks, food additions, and any material arriving in shipment containers. The large variation in both alpha and beta diversity between stores was therefore surprising. Samples collected from the same store generally clustered tightly together in PCA analyses (Figure 3), despite the fact that we collected samples from different tanks with highly variable species composition (Table 2). Furthermore, inter-store differences were always large, despite a shared water source for the Providence area. This strongly suggests that characteristics of the stores themselves – including cleaning regimes, fish supplier, filtration type or handling procedures influence the diversity and community structure of bacteria within their tanks. Unfortunately, these kinds of contextual environmental data are lacking, and therefore it is difficult for us to draw conclusions about the underlying water quality differences between our stores that may be reflective of differences in community composition. Further research including this type of metadata could shed light on which husbandry techniques pet stores use, or distributors fish are purchased from, facilitate healthy fishes and aquarium environments, and which do not.

### Potential Pathogens

Our survey identified 53 genera that contain potentially pathogenic species (Table S3) and eleven species known to cause disease in fishes, humans and other species (Table 2). However, 16S rRNA -based taxonomy does not provide the resolution necessary to distinguish between innocuous and virulent organisms, which can often have identical primary structure along much of the molecule. We can therefore only comment on the potential presence of a virulent strain within our samples, based on the presence of its higher taxonomy. This is why we refer to them as potential pathogens. It is important to note that the role mobile genetic elements play in the ecology of virulence is still poorly understood [25], but evidence exists that single-step transfers of large DNA fragments can happen rapidly within a species [26], potentially establishing virulence in a formally innocuous strain. It is therefore reasonable to assume that the detection of a pathogenic species, even if not resolved to the strain level, represents a potential disease risk.

It is beyond the scope of this paper to discuss each of these potential pathogens in depth though some context is warranted. Many of the species in Table 2 are generally considered to infect fishes or other animals opportunistically, and subsequent diseases typically develop in ornamental and aquaculture fishes when animals are stressed. Though rare, primary disease threats to humans from ornamental fishes often result from accidental ingestion of contaminated tank water or introduction of pathogenic bacteria through open wounds [27]–[29]. Common bacterial threats identified in the literature appear to be *Mycobacterium*, *Salmonella, Aeromonas* and *Legionella* infections in children, pregnant women and other immune compromised populations [29]–[32].

Among the potential fish pathogens identified in this survey, *Aeromonas* and *Vibrio* species pose the most significant threats to ornamental fishes. These bacteria are all common inhabitants of healthy fishes and aquatic systems that can become pathogenic and cause substantial mortality when conditions are stressful [33]–[35]. The release of pathogenic bacteria from stressed, morbid and dead fishes into carriage (during shipping) and aquarium water shared by other animals exacerbates the risk of disease in ornamental fish trade and is of specific concern to industry. Diseases these species cause may occur under normal tank conditions, however outbreaks are more common when fishes are stressed by low oxygen, high ammonia, high nitrate, high water temperature, rough handling, mechanical injury and generally over-crowding [33]–[34]. The genus *Aeromonas* includes several species that cause some of the most common bacterial infections in freshwater fishes that frequently induce external hemorrhages, distended abdomens and protruding eyes, and though mortality rates are typical low (<10%) many strains are resistant to commonly used antibiotics, making control for industry difficult [36]. *Aeromonas* outbreaks in ornamental fishes can almost always be tied back to poor water quality and rough handling. *A. shubertii, A. veroni* and *A. hydrophila* can cause wound infections in humans, gastroenteritis in healthy individuals, and opportunistic systemic disease in immune compromised individuals [36].

Vibrios are typically associated with marine and brackish environments but are occasionally detected in freshwater fishes and environments, as they were in this study. *Vibrio* infections can spread rapidly when fishes are confined in heavily stocked, commercial systems where morbidity may reach 100% in affected facilities [35], [37]. *V. vulnificus* is the most common fish-derived *Vibrio* infection in humans, with exposure resulting largely from puncture wounds and ingestion, and clinical signs manifesting as necrotizing fasciitis, edema, and swelling at the site of puncture [37]. *V. cholerae* is perhaps the most widely recognized of the *Vibrio* species, annually afflicting millions of people worldwide, primarily in tropical developing nations. *V. cholerae* infections result from ingestion of contaminated water or via infected shellfish and >100 million bacteria are required to cause disease in a healthy individual though this is far less in immune compromised populations and children. *Vibrios* have previously been identified in aquaria though no causes resulting in human disease have been reported [38]–[40]. In our study, the prevalence of *V. cholerae* among our detected *Vibrio* OTUs is noteworthy. *V. cholerae* (non-O1) has been detected in diseased goldfish [41], the common carp, cichlid, *Tilapia* and mullet [42]. While there are reports of vibrios causing disease in fishes, some reports suggest they may aid in fish digestion and are beneficial to the fish intestine [42].

### Future Directions

Our results, combined with evidence from the literature, suggest that ornamental fishes and aquarium tank water are an understudied system with highly diverse microbial communities and sources of potential pathogens of interest to the pet industry and public health. Many of the potentially pathogenic bacteria discovered in our survey cannot be eradicated as they are part of the normal microbial flora of myriad hosts and aquatic environments. And, as described above, they are not always harmful. Nevertheless, risks exist and so we encourage owners of ornamental fishes and the pet industry to take responsibility for the health of the animals in their care and the people caring for them. Risk reduction can benefit from additional science aimed at providing a deeper understanding of the microbial ecology of aquarium systems and especially the industry/consumer practices that influence microbial community diversity and facilitate opportunistic infections. Such knowledge can be distilled into specific consumer and industry outreach initiatives. Guidelines have been established to help prevent salmonellosis in reptile owners (see those from the Association of Reptilian and Amphibian Veterinarians and the Centers for Disease Control) and help industry eliminate pathogen-carrying ticks on reptiles imported to the U.S. for sale in the pet trade (PIJAC's National Reptile Improvement Plan). Similar agendas may be created for ornamental fishes, perhaps in line with the Marine Aquarium Council's certification program. Consumer education initiatives on the topic of healthy pets are already reaching more groups (i.e. PetWatch and CDC's Healthy Pets Healthy People), some of which include information on ornamental fishes. After a series of failed policy attempts to address disease in wildlife trade [3], a multi-pronged approach that unites consumers, industry and scientists to reduce potential pathogens and disease in the nation's pet population, ornamental fishes included, seems to be the most realistic way forward.

## Methods

### Sites, Species and Sample Collection

Over a two-day period in November 2009, we purchased freshwater common goldfish (*Carassius auratus*) and Chinese algae eaters (*Gyrinocheilus aymonieri*) from seven pet stores in the Providence area of Rhode Island. Two stores represented national chains and five were locally-owned small businesses. We purchased two individuals of each species at six stores and an additional two Chinese algae eaters at one store. Each individual was associated with a single tank (resulting in 14 tanks sampled). Store employees collected fishes and water and bagged individuals of a single species together (two individuals per bag) with ∼300–500 mL of tank water. We immediately transported bags to Brown University in Providence, RI for processing.

We manually filtered water samples to concentrate microbial biomass immediately upon arrival at the lab. Sterile 60 mL syringes were used to transfer water directly from the plastic bags onto 0.2-µm Sterivex filter units (Millipore, Billerica, MA). We filtered a total of 600 mL of water per bag such that each filter corresponded to a single tank in a single store, yielding 14 filtered samples. Air pushed through each filter three times served to remove any residual water. After filtration was complete, we placed filter cartridges immediately on dry ice and stored them frozen at −80°C until transport on dry ice to the MBL at Woods Hole for further processing. Following sampling, one of the authors kept the fishes as personal pets.

### DNA Extraction

DNA extraction followed Puregene (Qiagen, Valencia, CA) kit instructions with the following modifications. We removed the filter inside of the sterivex using a sterilized pvc pipe cutter. We then used a sterile razor blade to cut the filter into two halves and placed each half into a screw-cap tube containing Puregene lysis buffer. Cell lysis was accomplished via the addition of lytic enzyme and proteinase K incubation followed by bead beating with 0.1 mm zirconium beads (Biospec products #11079101z). We bead-beated the cells at 5000 rpm for 60 seconds using a Beatbeater 8 (Biospec Products, Bartlesville, OK). The remainder of the protocol followed the manufacturer's instructions. Water filtration and DNA extraction protocols are available for download at http://amarallab.mbl.edu.

### PCR-screening and Cloning of Potential Pathogens

We used diagnostic PCR primers to determine the presence or absence of 9 bacterial and 4 eukaryotic genera that contain common human pathogens across our 14 freshwater aquarium tank samples from the 7 surveyed pet stores. We based our primer selection on previously published reports or personal communication and targeted bacterial 16S rRNA gene, eukaryotic 18S rRNA gene, or protein-coding genes involved in pathogenicity (see Table S2 for details of primers and citations). We confirmed the quality of the template DNA for PCR by performing bacterial 16S rRNA gene using general primers 27F and 1492R and eukaryotic 18S rRNA gene amplifications using universal EukA and EukB primers targeting the 5′ and 3 ends of the 18S rRNA gene respectively [43].

Amplifications employed the Phusion High-Fidelity PCR kit (Finnzymes, Espoo, Finland) at 98°C denaturation for 1 minute followed by 25 cycles at 98°C for 5 seconds, primer annealing temperature for 15 seconds, and 72°C for 30 seconds, followed by a final 5 minutes at 72°C. Annealing temperature varied depending on the melting temperature (T_m_) of each primer set, but was generally 3°C above the lowest primer T_m_. An amplification was labeled “negative” only after multiple failed amplifications, but we acknowledge that the lack of amplification is not conclusive proof of absence. For nested PCRs, outside amplifications ran under the same conditions but employed 5 fewer cycles.

We used the TOPO® cloning kit with Mach1™-T1^R^ *E. coli* strain chemically competent cells (Life Technologies, Carlsbad, CA) to clone PCR products following manufacturer's protocols. We sequenced cloned PCR products on an Applied Biosystems 3730XL capillary sequencer, and edited resulting reads using an in-house script to remove vector sequences and low quality base calls. Alignments of forward and reverse sequences, and sequence proofreading were done manually in Geneious ver. 5.4 Software [44]. We assessed taxonomic assignments using the BLAST search algorithm [45]. Sequences and MIMARKS compliant metadata were deposited in the National Center for Biotechnology Information's (NCBI) GenBank under accession numbers JX317526 - JX317619.

### Amplicon Sequencing

Pyrosequencing methodologies for 16S rRNA gene amplicon sequencing have been described previously [7], [8], [12], [46] and were performed on 2 samples each from stores A, D and E. Briefly, we amplified the bacterial 16S rRNA gene hypervariable region spanning the V3–V5 region in triplicate using a cocktail of 2 forward primers at the *E. coli* 16S rRNA gene position 341, and a cocktail of three reverse primers at position 926 (Table S1), yielding amplicons ∼585 base pairs in length. We multiplexed our sequencing reactions by using primers with an in-line 5-bp barcode between the primer and the 19 nt Roche 454 A adaptor [8], [12]. Amplicons and negative controls were spin-column purified using QIAquick PCR purification kit (Qiagen, Valencia, CA) and sizes were confirmed on a Bioanalyzer 2100 (Agilent, Palo Alto, CA) using a DNA1000 LabChip. Purified amplicons were then brought through emPCR and sequenced on a Roche GS-FLX pyrosequencer using GS FLX Titanium Series reagents (Roche Diagnostics, Basel, Switzerland) following manufacturer's protocols.

We also performed separate pyrosequencing reactions using *Vibrio-*specific primers on 2 samples each from stores A, D and E, and a single sample from store B. Note that this run included a single sample from a store (B) not included in the V3–V5 run. This run was intended to deeply sample *Vibrio* diversity but resulting amplicon taxonomy assignments fell broadly within the Gammaproteobacteria. Protocols for this run were identical to those described above, except primers targeted the 518F and 680R regions of *E. coli* (∼120 nt) and were run using FLX reagents on the Roche 454- GS-FLX. MIMARKS-compliant sequence data have been deposited in NCBI's normal and Sequence Read Archives (SRA) under the accession number SRP013874, the associated metadata can also be found in Table S4.

### Bioinformatics

We processed raw reads through the VAMPS pipeline [46], and took the following quality control measures for GS-FLX titanium. We removed reads if any of the following were true: (1) we detected sequence mismatches to the expected 5-nt barcode or proximal primer, (2) we observed an ambiguous base call (N) anywhere in the read, (3) we could not find a match to the conserved region used to trim all sequences to the same position in 16S rRNA gene alignment (5′-CCCATAGATTAGG-3′), (4) if the trimmed length was below 375 nt, (5) if the average quality score was below 30, (6) if the read was not identifiable by GAST as having a percent identity of at least 70% to a known bacterial sequence and, (7) if the read contained an gap or deletion in the alignment to the nearest reference sequence of 10 nt or more. Chimeras were removed using UCHIME [47] and 3% OTUs were assigned using UCLUST as implemented in USEARCH v 5.1 [47]. Global Alignment Sequence Taxonomy (GAST) algorithms assigned taxonomy to the most abundant read within an OTU as described previously [12]. Briefly, each sequence that completed the trimming and filtering steps was subjected to a BLAST search against a local database created from high quality reads from the SILVA-ARB archive [48]. The sequenced tag was then aligned with MUSCLE [49] against its top 100 BLAST hits and the GAST distance to each hit was calculated by adding the number of insertions, deletions and mismatches over the total length of the tag. All sequences from the reference database were then queried for exact matches to the top GAST hit (not necessarily the top BLAST hit), and the RDP taxonomic classification of these exact matches were returned. If two thirds of the classifications were the same taxonomic ID, then that taxonomy was assigned to that tag.

We calculated alpha diversity using both phylogenetic diversity (PD) and best-fit parametric based models using CatchAll [20]. Prior to phylogenetic diversity calculation we resampled data such that all samples had equal sampling effort. Rarefaction randomly subsamples species abundance tables down to the lowest number among all samples, thus removing heterogeneity between samples [50], [51]. Phylogenetic diversity was then calculated as the minimum total length of the phylogenetic branches required to span all taxa within a given sample on a phylogenetic tree [52]. Since all sequences from a study are placed in the tree, this estimate is not influenced by the particularities of sequence clustering algorithms. We performed both rarefaction and phylogenetic diversity estimates in Qiime v1.4.0 [19] using the PD Whole Tree estimator.

Our phylogenetic diversity estimates showed strong evidence for inter-store differences, however PD estimates are descriptive, sample based only and do not allow extrapolation to a population. To provide this additional context, we also calculated alpha diversity using CatchAll 3.0 [20]. CatchAll computes a large range of finite-mixture models and all known nonparametric and parametric coverage-based estimates, and presents the model which best fits each dataset, or the ‘best of the best’ fit model. It also provides standard errors, goodness of fit and confidence intervals for each estimate [20].

### Visualizing *Vibrio* Diversity

Although the GAST strategy provides an efficient way to assign taxonomy to our OTUs, it is quite conservative. To further refine *Vibrio* taxonomy, we constructed a reference tree using selected full-length *Vibrio* sequences from published isolates in the Silva ARB 16S SSU_ref_102 rRNA database. To this reference tree we added any OTU representative sequences returned from GAST with at least a Vibrionaceae taxonomic assignment (24 OTUs representing 12,175 sequences) using ARB's quick-add-sequence-to-tree parsimony. This method allowed us to visualize the diversity of our *Vibrio* OTUs independent of the GAST assignments (Figure 4).

## Supporting Information

Table S1 **Diagnostic PCR primers used to determine presence of potential pathogenic genera, and to clone** ***Legionella*** **,** ***Vibrio*** **and** ***Aeromonas*** **.**
(DOCX)

Table S2 **Sequence of universal bacterial primer cocktail used to amplify V3–V5 16S rRNA gene hypervariable region.**
(DOCX)

Table S3 **List of genera found from V3–V5 454 run.** List was compiled by comparing GAST genus level taxonomy of our sequences to all known bacterial human pathogens as found in Taylor et al. 2001 data supplement. Any genus matches were included in this list.
(DOCX)

Table S4 **MIMARKS table of associated sample metadata.**
(XLSX)
