# Supplementary material for: Microbial Diversity and Potential Pathogens in Ornamental Fish Aquarium Water
Source: PLoS One. 2012 Sep 6;7(9):e39971. doi: 10.1371/journal.pone.0039971 (PMC3435374; doi:10.1371/journal.pone.0039971)
Supplement: Table S1 — Diagnostic PCR primers used to determine presence of potential pathogenic genera, and to clone Legionella , Vibrio and Aeromonas . (DOCX) [file pone.0039971.s001.docx]

| **Taxon** | **#positive/**  **#tested** | **Primer Target** | **Citation** |
| --- | --- | --- | --- |
| *Legionella* | 12/14 | 16S rRNA gene | Jonas et al. 1995 |
| *Acanthamoeba* | 0/10 | 18S rRNA gene | Gast et al. 2001 |
| *Giardia* | 0/11 | Heat Shock Protein | Abbaszadegan et al. 1997 |
|  |  | 18S rRNA gene | Lasek-Nesselquist, unpublished |
| *Francisella* | 0/10 | 16S rRNA gene | Barns et al. 2005 |
| *Cryptosporidium* | 4/10 | 18S rRNA gene | Ward et al. 2002 |
| *Campylobacter* | 0/14 | Flagellin gene | Waage et al. 1999 |
| *Vibrio* | 11/14 | 16S rRNA gene | Thompson et al. 2004 |
| Corynebacterineae | 5/14 | 16S rRNA gene | de los Reyes et al. 1997 |
| *Mycobacterium* | 10/14 | 16S rRNA gene | de los Reyes et al. 1997 |
| *Aeromonas* | 7/14 | 16S rRNA gene | Franke-Whittle et al. 2005 |
| *Naegleria* | 0/14 | ITS region | Pélandakis et al. 2000 |
| *Salmonella* | 0/14 | Pathogenicity Island I | Loy et al. 2007, ProbeBase |
| Universal Bacteria | 14/14 | 16S rRNA gene | Lane 1991 |
| Universal Eukarya | 10/10 | 18S rRNA gene | Medlin et al. 1998 |
