# Supplementary material for: Microbial Diversity and Potential Pathogens in Ornamental Fish Aquarium Water
Source: PLoS One. 2012 Sep 6;7(9):e39971. doi: 10.1371/journal.pone.0039971 (PMC3435374; doi:10.1371/journal.pone.0039971)
Supplement: Supplementary file 2 [file pone.0039971.s002.docx]

Primer ID	Roche A Adaptor	Barcode	Universial Bacterial Primer Cocktail
341F2	GCCTTGCCAGCCCGCTCA	NNNNN	CCTACGGGNGGCWGCAG
341F3	GCCTTGCCAGCCCGCTCA	NNNNN	TCTACGGAAGGCTGCAG
926R1	GCCTTGCCAGCCCGCTCA	NNNNN	CCGTCAATTCNTTTRAGT
926R3	GCCTTGCCAGCCCGCTCA	NNNNN	CCGTCAATTTCTTTGAGT
926R4	GCCTTGCCAGCCCGCTCA	NNNNN	CCGTCTATTCCTTTGANT
