# Supplementary material for: Microbial Diversity and Potential Pathogens in Ornamental Fish Aquarium Water
Source: PLoS One. 2012 Sep 6;7(9):e39971. doi: 10.1371/journal.pone.0039971 (PMC3435374; doi:10.1371/journal.pone.0039971)
Supplement: Supplementary file 3 [file pone.0039971.s003.docx]

Legionella	*Chryseobacterium*	*Halomonas*	*Pseudomonas*
Acinetobacter	*Citrobacter*	*Janthinobacterium*	*Psychrobacter*
Aeromonas	*Clostridium*	*Klebsiella*	*Rhodococcus*
Alteromonas	*Corynebacterium*	*Lactococcus*	*Rickettsia*
Aquaspirillum	*Coxiella*	*Leptospira*	*Serratia*
Arcobacter	*Cytophaga*	*Moritella*	*Shewanella*
Arthrobacter	*Ehrlichia*	*Mycobacterium*	*Simkania*
Bacillus	*Enterobacter*	*Neisseria*	*Staphylococcus*
Bordetella	*Enterococcus*	*Nocardia*	*Streptococcus*
Brevibacterium	*Erysipelothrix*	*Orientia*	*Tatlockia*
Burkholderia	*Flavobacterium*	*Photobacterium*	*Tenacibaculum*
Chlamydophila	*Flexibacter*	*Piscirickettsia*	*Treponema*
Chromobacterium	*Francisella*	*Plesiomonas*	*Vibrio*
